# Locally Estimated Scatterplot Smoothing for Censored Survival Data

**DOI:** 10.1002/sim.70783

**Published:** 2026-10-17

**Authors:** David van Klaveren, Peter C. Austin, Patrick W. Serruys, Ben van Calster, Frank E. Harrell

**Affiliations:** ^1^ Department of Public Health Erasmus University Medical Center Rotterdam Rotterdam the Netherlands; ^2^ Predictive Analytics and Comparative Effectiveness Center Institute for Clinical Research and Health Policy Studies, Tufts Medical Center Boston Massachusetts USA; ^3^ ICES Toronto Ontario Canada; ^4^ Institute for Health Policy, Management and Evaluation, University of Toronto Toronto Ontario Canada; ^5^ Sunnybrook Research Institute Toronto Ontario Canada; ^6^ CORRIB Research Centre for Advanced Imaging and Core Laboratory University of Galway Galway Ireland; ^7^ Department of Development and Regeneration KU Leuven Leuven Belgium; ^8^ Leuven Unit for Health Technology Assessment Research (LUHTAR) KU Leuven Leuven Belgium; ^9^ Julius Center for Health Sciences and Primary Care University Medical Center Utrecht Utrecht the Netherlands; ^10^ Department of Biostatistics Vanderbilt University School of Medicine Nashville Tennessee USA

**Keywords:** calibration curve, censoring, LOESS, prediction models, survival data, validation

## Abstract

In the absence of censoring, locally estimated scatterplot smoothing (LOESS) is a powerful tool to estimate the relationship between a continuous covariate and the probability of an event and is often used to visually assess calibration of prognostic models. Since follow‐up in prognostic studies is often incomplete, we aimed to study the extension of LOESS to right‐censored data. In line with other work, we first obtained inverse‐probability‐of‐censoring (IPC) weights through Kaplan–Meier estimation. When censoring varied by covariate levels, we used Cox regression with a spline function of the covariate to obtain IPC weights. We then applied LOESS to the weighted event indicators to estimate the relationship between the covariate and the probability of an event within a specific time horizon. We illustrated the application of LOESS for censored data (LOESScd) using patients with complex coronary artery disease. We used the LOESScd to estimate the association between continuous predictors and the probability of death within 10 years. LOESScd estimates closely followed grouped Kaplan–Meier estimates, especially in case of weak smoothing. Covariate‐dependent IPC weights improved LOESScd when censoring varied by covariate levels. We then examined the finite‐sample behavior of LOESScd in simulations motivated by four typical prognostic model validation scenarios. In 2000 samples of 1800 patients, bias was limited and coverage was close to nominal. In conclusion, LOESScd is a promising estimator of the relationship of a continuous covariate with the probability of an event in the presence of right‐censoring. LOESScd is especially useful for estimating calibration curves when validating prognostic models.

## 1 Background

In the absence of censoring—when events are fully observed—nonparametric locally estimated scatterplot smoothing (LOESS) is a powerful tool to estimate the relationship between a continuous covariate and the probability of an event within a specific time horizon, for example, the relationship between age and 5‐year mortality in patients with coronary artery disease [1]. LOESS is also often used to visualize the agreement between model predictions and the probability of an event when assessing the calibration of prognostic models for a fully observed binary outcome [2, 3]. LOESS fits a smooth curve to bivariate data, by using weighted least squares regression on local neighborhoods of the data. Data points closer to the point of interest are weighed more heavily. The proportion of data points considered in local neighborhoods (“span”) determines the strength of smoothing (large span = strong smoothing). LOESS allows data analysts to visualize complex relationships between two variables where parametric methods may fall short, especially when the underlying relationship is not easily captured by a global model. LOESS is considered one of the most attractive modern regression methods for nonparametric data visualization and is implemented in many statistical software packages (R; Python; SAS; SPSS), but is not directly applicable to right‐censored data [4].

Analysis of right‐censored data is common in medical and epidemiological research. A familiar example is analyzing time‐to‐death in clinical trials, where many patients are still alive at the end of the trial [5]. Techniques have long been available for estimating the probability of an event within a specified time horizon for groups of subjects (Kaplan–Meier estimates) [6], and for estimating a parametric relationship between a continuous covariate and the probability of an event within a specified time horizon, for example, using a spline transformation of the continuous covariate in a Cox or hazard regression [7, 8]. Furthermore, learning techniques have been adapted for use in right‐censored data on the basis of censoring unbiased transformations of continuous outcomes such as (log) survival times [9, 10, 11, 12]. For application of binary classification techniques, a reweighing of the training dataset has been proposed with inverse‐probability‐of‐censoring (IPC) weights based on the reverse Kaplan–Meier estimator [13].

In a prognostic modeling context, we have previously proposed to estimate a calibration curve of a prognostic model using Cox regression with a restricted cubic spline to specify the relationship between model predictions (on the log‐hazard scale) and the hazard of right‐censored outcomes [14]. However, even semiparametric estimates typically rely on global model assumptions, such as proportional hazards or restricted cubic splines to transform the continuous covariate. Therefore, a nonparametric estimator may be preferable, giving better estimates than (semi)parametric alternatives in scenarios where (semi)parametric model assumptions are violated. Symmetric nearest neighbor smoothing of pseudo‐observations has been proposed as a non‐parametric alternative which requires that the censoring distribution is independent of the covariate of interest (the predicted probability in a calibration curve) [3, 15].

We aimed to study a straightforward extension of LOESS to estimate a smooth relationship between a continuous covariate and the probability of an event within a specified time horizon in the presence of random or covariate‐dependent right‐censoring. We illustrated locally estimated scatterplot smoothing for censored data (LOESScd) in case study data with varying censoring mechanisms and examined the finite‐sample behavior of LOESScd in simulations motivated by four typical prognostic model validation scenarios.

## 2 Methods

### 2.1 LOESS for Censored Data

In line with other work, we will use IPC weights to transform the observed outcomes, but we will focus on the survival probability at a specific timepoint and directly multiply the IPC weights with the events observed before that time point [9, 10, 11, 12, 13]. This straightforward use of IPC weights is intuitively related to the fact that the nonparametric Kaplan–Meier estimator at a fixed time point can be written as a weighted average of the events observed before that time point, with weights equal to the inverse probability of observing the event [16]. At a specific covariate level $x^{\prime }$—or in a small neighborhood of covariate level $x^{\prime }$—such a weighted average of observed events is an estimator of the probability of the event conditional on $x=x^{\prime }$. To estimate a smooth relationship between a continuous covariate x and the probability of the event, we use the LOESS of the relationship between observations of x and the weighted events.

For i = 1, …, N let $T_{i}^{*}$ be the random variable denoting the (possibly unobserved) failure time and $C_{i}$ be the random variable denoting the (possibly unobserved) censoring time for subject i. Let $T_{i}=\min\left(T_{i}^{*},C_{i}\right)$ and let $\Delta_{i}=I\left(T_{i}^{*}\leq C_{i}\right)$. The data typically consist of observed pairs of $\left(T_{i},\Delta_{i}\right)$, denoted as $\left(t_{i},\delta_{i}\right)$. The Kaplan–Meier estimator $\hat{S}\left(t\right)$ for the survival probability at time t is usually written as a product‐limit estimator [6], but can also be written as an IPC weighted average of the observed events at time t: [16] $\hat{S}\left(t\right)=1-\frac{1}{N}\sum_{i=1}^{N}\frac{I\left[t_{i}\leq t\right]\delta_{i}}{\hat{K}\left(t_{i}-\right)}$. With $\hat{K}\left(t_{i}-\right)$ the Kaplan–Meier estimate of $\mathrm{Pr}\left(C_{i}\geq t_{i}\right)$, the probability that a failure time is observed. The “reverse Kaplan–Meier estimate” $\hat{K}\left(t_{i}-\right)$ is easily calculated by treating failure events as “censored observations” and censored observations as “failure events.” Note that the summands are either 0 (for censored observations at time t) or the inverse probability of observing the failure time $\left(\frac{1}{\hat{K}\left(t_{i}-\right)}\right)$ for failure events before time t. The central idea of this paper is that we can directly apply standard locally estimated scatterplot smoothing to the summands $\frac{I\left[t_{i}\leq t\right]\delta_{i}}{\hat{K}\left(t_{i}-\right)}$ for estimating a smooth relationship between a continuous covariate and the probability of failure before time t. When we are interested in the probability of survival beyond time t rather than failure before time t, we can use one minus the smooth estimator described above.

The estimator introduced here is based on Kaplan–Meier estimates of the probability that a failure time is observed. Consequently, these estimates express the probability that a failure time is observed for the whole sample, regardless of the level of covariate x. When censoring is likely to depend on x, we can alternatively estimate the probability $\hat{K}\left(t_{i}-;x_{i}\right)$ that the failure time of patient i is observed conditional on $x_{i}$, for example using Cox regression with x or a (spline) function of x as covariate, with the requirement that $\hat{K}\left(t_{i}-\right)>0$ for failure times observed before time t. This feature is implemented in the LOESScd *R* function which we will use in the remainder of this paper (Box 1).

BOX 1 **LOESScd function in R programming language.**

# *x* is a continuous variable.

# S is a Surv object (Survival package [32]).

# *u* is the time point of interest.

# span is the smoothing span with default 0.75.

# formula.cens is the model for the censoring probability with sample‐based Kaplan–Meier.

# estimates as default (∼1). Alternatively, a Cox model can be used, for example, with a linear.

# relationship between *x* and the log‐hazard of censoring (∼*x*) or a spline relationship.

# between *x* and the log‐hazard of censoring (∼pspline(*x*)).

loess.surv ⟵ function(*x*, *S*, *u*, span = 0.75, formula.cens = ∼1)

{

**Table:** 

*y* ⟵ *S*[,1]	# Obtain event times
*e* ⟵ *S*[,2]	# Obtain event indicators
*y*.*u* ⟵ pmin(*y*,*u*)	# Observed event times at time *u*
*e*.*u* ⟵ *e* * (*y*<*u*)	# Event indicators at time *u*
*e*.cens.*u* ⟵ (1‐*e*) * (*y*<*u*)	# Indicators of censoring at time u
*G* ⟵ coxph(formula(paste("Surv(*y*.*u*, e.cens.*u*)", paste(formula.cens, collapse = ""))))	# Fit censoring model G
*K* ⟵ exp(−predict(*G*,type = ‘expected’))	# Probabilities that event times are observed
*e*.loess ⟵ *e*.*u* * 1/*K*	# The event indicators are inflated with 1/*K*
loess.surv ⟵ loess(*e*.loess˜*x*, span = span)	# Standard loess with inflated event indicators

}

### 2.2 Case Study

The SYNTAX Extended Survival (SYNTAXES; NCT03417050) study is an investigator‐driven extension follow‐up of a multicenter, randomized controlled trial (SYNTAX; NCT00114972) done in 85 hospitals across 18 North American and European countries between March 2005 and April 2007 [17, 18]. Patients (*n* = 1800) with de‐novo three‐vessel and left main coronary artery disease were randomly assigned to either percutaneous coronary intervention (PCI) or coronary artery bypass grafting (CABG). The SYNTAXES study ascertained 460 10‐year all‐cause deaths.

The SYNTAX Score 2020 was developed in the SYNTAXES data to predict 10‐year mortality, conditional on the chosen revascularization strategy [19]. The SYNTAX Score 2020 includes seven prognostic factors for mortality (age; creatinine clearance; left ventricular ejection fraction; chronic obstructive pulmonary disease; peripheral vascular disease; medical treatment of diabetes; current smoking) and two treatment‐covariate interactions, with disease type (three‐vessel disease or left main coronary artery disease) and with the anatomical complexity of the disease (expressed by the anatomical SYNTAX score).

We first used LOESScd to visualize the association between patient age and the probability of death within 10 years in the original SYNTAXES data. Since the proportion of loss‐to‐follow‐up was limited in the SYNTAXES data (0.074 at 10 years), we also visualized the association between age and the probability of death within 10 years in the SYNTAXES data with additional censoring. To do so, we randomly added exponentially distributed censoring such that the probability of censoring within 10 years was 0.50. In a subsequent analysis, we added exponentially distributed censoring such that the probability of censoring within 10 years increased linearly from 0 at age 30 to 0.50 at age 80. In each of these datasets, we applied two versions of LOESScd. The first LOESScd estimate assumed random censoring—with the probability of censoring based on the reverse Kaplan–Meier estimator. The second LOESScd estimate allowed for possible age‐dependent censoring—with the probability of censoring based on a Cox model including a penalized spline of age with four degrees of freedom [20].

Second, we used LOESScd in the original SYNTAXES data to visualize the association between the probability of death within 10 years and the other three continuous covariates that were used to develop the SYNTAX Score 2020: anatomical SYNTAX Score; creatinine clearance; and left ventricular ejection fraction.

Third, we used LOESScd to estimate calibration curves in patients who were assigned to PCI and CABG, respectively. These calibration curves visualize the agreement between predicted 10‐year mortality according to the SYNTAX Score 2020 and observed 10‐year mortality. We estimated the calibration curves in the original SYNTAXES data as well as in the SYNTAXES data with additional random censoring.

### 2.3 Simulation Study

We used simulations to examine the finite‐sample behavior of LOESScd when it is used for estimating calibration curves in an external validation of a prognostic model. To do so, we compared estimates of calibration curves with true calibration curves—known because we control the true data generation process—in four common scenarios that occur when validating prognostic models in practice: perfect calibration; overestimation; linear overfitting; and nonlinear miscalibration.

We used the SYNTAXES data as the basis for the generation of a representative superpopulation (*n* = 100 000). We first generated nine covariates which are the predictors of the SYNTAX Score 2020: four normally distributed continuous covariates (age; anatomical SYNTAX score; creatinine clearance; and left ventricular ejection fraction) and five Bernoulli distributed covariates (smoking status, and whether patients have medically treated diabetes, are receiving insulin, have chronic obstructive pulmonary disease, and have peripheral vascular disease). To preserve the correlations between covariates, we used the BinNor R package with the observed covariance matrix as input [21, 22].

We generated time‐to‐event outcomes from a Weibull distribution with a shape parameter of 1.09 that was estimated from the original SYNTAXES data. The scale parameters λ=exp(α+f(lp)) were derived from the linear predictors lp of the SYNTAX Score 2020 and a constant α according to four typical scenarios: (1) “Perfect calibration” where outcomes are generated with f(lp)=lp and α such that the generated 10‐year mortality is on average equal to the predicted 10‐year mortality with SYNTAX Score 2020; (2) “Overestimation” with f(lp)=lp and α such that the generated 10‐year mortality is on average 0.05 lower than predicted with SYNTAX Score 2020; (3) “Linear overfitting” where f(lp)=0.8lp and α such that the generated 10‐year mortality is on average equal to the predicted 10‐year mortality with SYNTAX Score 2020; and (4) “Nonlinear miscalibration” where the linear predictors are transformed to $f\left(\mathrm{lp}\right)=\frac{1}{2}\mathrm{lp}+\frac{1}{18}{\left(\mathrm{lp}+1\right)}^{3}$ and α such that the generated 10‐year mortality is on average equal to the predicted 10‐year mortality with SYNTAX Score 2020.

We generated censoring times according to two scenarios: exponentially distributed censoring times such that the probability of censoring within 10 years was 0.50; and exponentially distributed censoring such that the probability of censoring within 10 years increased linearly from 0.30 to 1 over the full range of predicted 10‐year mortality (0 to 1).

In 2000 randomly drawn samples of 1800 patients we used the LOESScd to estimate 10‐year mortality as a function of the predicted 10‐year mortality with 0.95 confidence bands. We assessed bias and coverage of these estimates at 200 equidistant quantiles of the distribution of true 10‐year probability of death in the superpopulation [23]. Bias is summarized by the mean, 0.75 and 0.95 quantiles and the maximum of the absolute bias across the 200 quantiles of the true 10‐year mortality distribution. The bias at each quantile of the true 10‐year mortality distribution is the difference between the true value and the mean of the estimates across 2000 samples. Coverage is summarized as the proportion of 0.95 confidence band estimates that cover the true value across 200 quantiles and 2000 samples.

## 3 Results

### 3.1 Case Study

LOESScd assuming random censoring was able to accurately visualize the association between patient age and the probability of death within 10 years in the original SYNTAXES data, with estimates that closely followed the grouped Kaplan–Meier estimates (Figure 1A). Based on the default smoothing span (0.75), LOESScd provided a more stable estimate of the association, while with weaker smoothing (smoothing span 0.25), local trends of the grouped Kaplan–Meier estimates were followed more closely, at the risk of overfitting to the data sample. The probability of censoring was very limited in the SYNTAXES data (weighted events are close to 1). A large proportion of loss‐to‐follow‐up (0.44) was concentrated around year 5, when the original SYNTAX study was extended to the SYNTAXES study, resulting in higher weights for events that occurred after year 5. This phenomenon is visualized by the two separate levels of weighted events (gray dots) in Figure 1A. When we used a spline function of age to model the probability of censoring, the weighted events (and the resulting LOESScd) were very similar, indicating that the relationship between age and loss‐to‐follow‐up was weak (Figure 1B).

**FIGURE 1:**
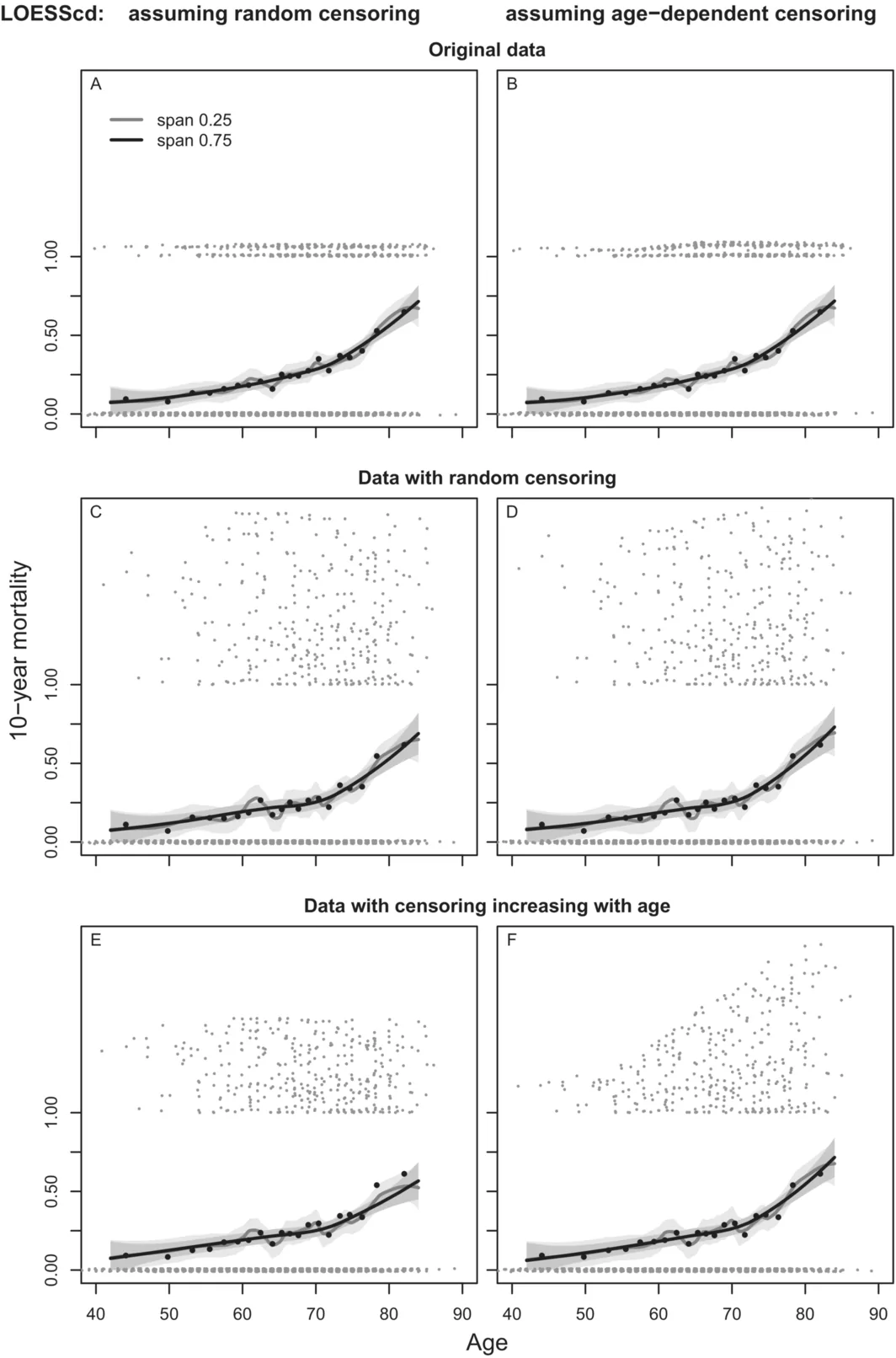
LOESScd estimates of the association between age and 10‐year mortality. Estimates in the original SYNTAX trial data (first row), with additional random censoring (second row), and with censoring that increases with age (third row). The estimates assume either random censoring (left column) or age‐dependent censoring (right column). The *y*‐value of the scatter is 0 for non‐events and the “inverse probability of observing the event time” for events, with small random perturbations to enhance visibility (gray dots). Black dots represent Kaplan–Meier estimates in 20 equally sized groups of increasing age. The shaded areas are 0.95 confidence bands of the LOESScd estimates.

With additional random censoring (0.50 at 10 years), LOESScd still visualized the association between patient age and the probability of death within 10 years very well, regardless of the censoring model (Figure 1C,D). Due to the additional censoring, the confidence bands of the LOESScd estimates were wider. When censoring was assumed random, but the actual censoring increased with age (Figure 1E), LOESScd estimates deviated from the grouped Kaplan–Meier estimates, especially at low and high age. With a spline function of age to model the censoring probability—nicely reflected by the increase of the weighted events with age in Figure 1F—the LOESScd estimates were again close to the grouped Kaplan–Meier estimates.

LOESScd estimates (assuming random censoring) of the association between the probability of death within 10 years and the continuous covariates that were used to develop the SYNTAX Score 2020 indicated different non‐linear patterns (Figure 2): The 10‐year probability of death increased more rapidly with increasing age when age was above 70 years compared to when age was below 70 years; the relationship with anatomical SYNTAX Score was fairly linear; and the 10‐year probability of death decreased with increasing Creatinine clearance and increasing LVEF, but less rapidly at higher values of these covariates.

**FIGURE 2:**
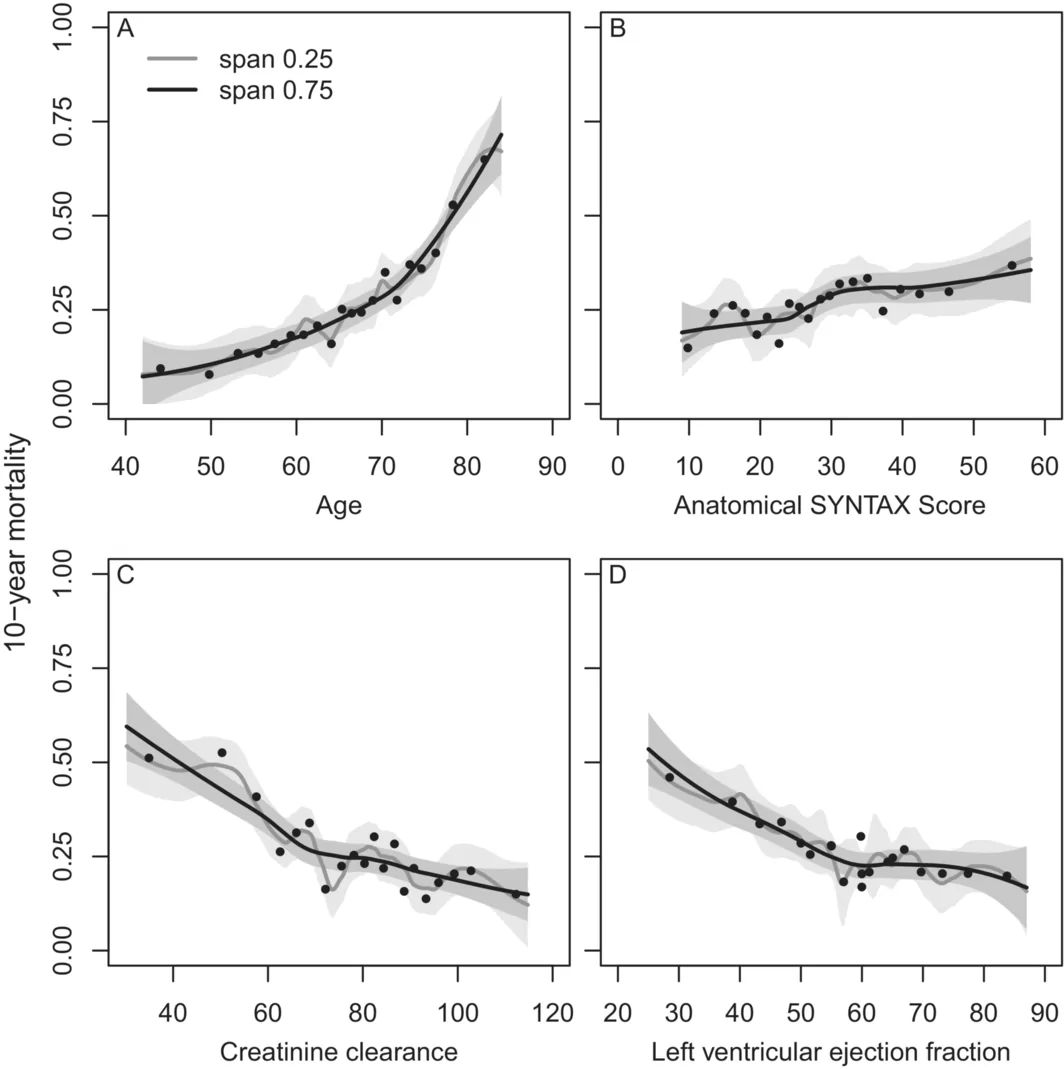
LOESScd estimates of the association between four continuous predictors and 10‐year mortality. Estimates of the association with 10‐year mortality of respectively age (Panel A), anatomical SYNTAX Score (Panel B), Creatinine clearance (Panel C) and Left ventricular ejection fraction (Panel D), in the original SYNTAXES trial data. Black dots represent Kaplan–Meier estimates in 20 equally sized groups. The shaded areas are 0.95 confidence bands of the LOESScd estimates.

When the calibration of SYNTAX Score 2020 was assessed in patients who were assigned to PCI and CABG separately (Figure 3A,B), the Kaplan–Meier estimates grouped by deciles of risk and the LOESScd estimates of the calibration curves indicated good agreement between predicted and observed 10‐year mortality. As expected, the grouped Kaplan–Meier estimates and the LOESScd estimates were slightly different and less precise when additional random censoring was added (Figure 3C,D), but the calibration was similar.

**FIGURE 3:**
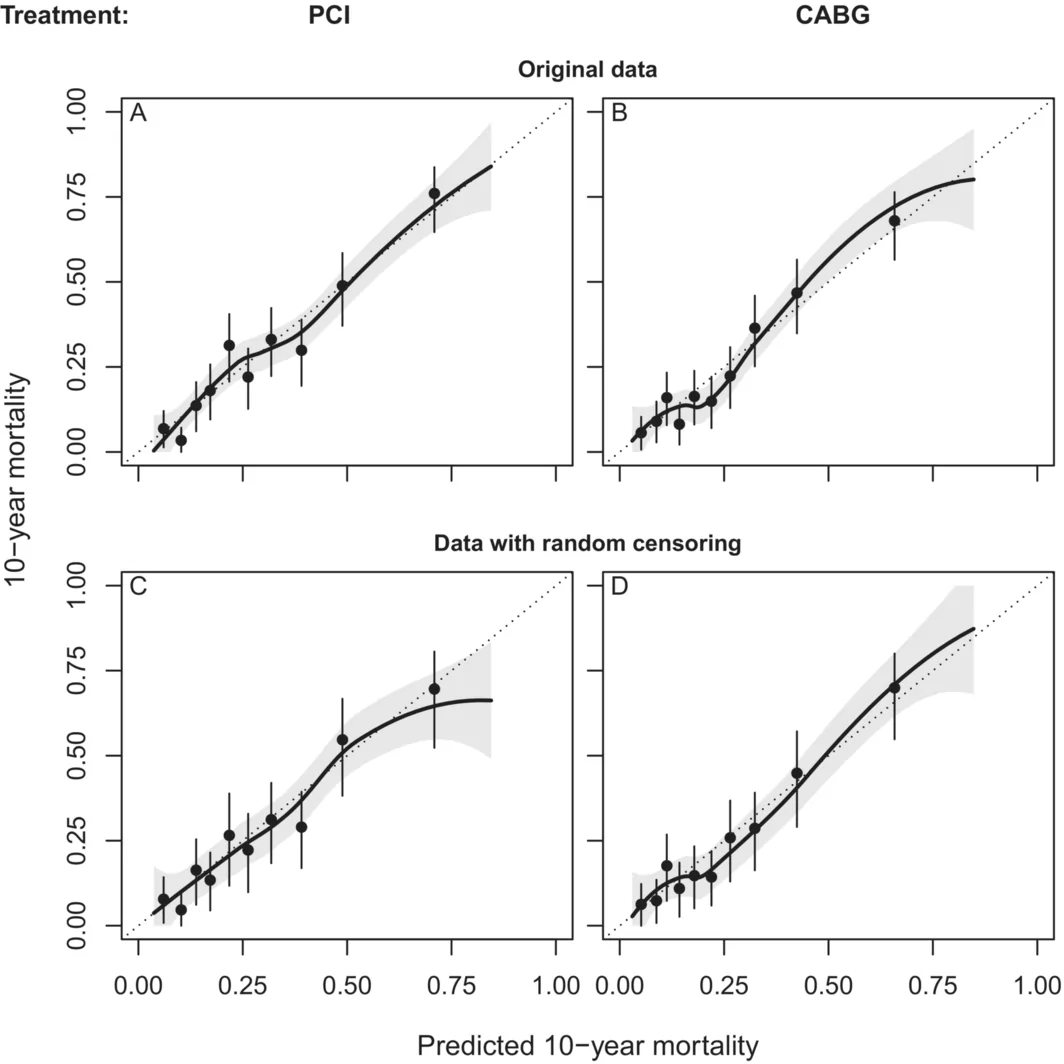
Calibration curves of 10‐year mortality predictions according to the SYNTAX Score 2020. Calibration curves are LOESScd estimates of the association between predicted 10‐year mortality according to the SYNTAX Score 2020 and observed 10‐year mortality. The calibration is measured in the PCI‐arm (left column) and in the CABG‐arm (right column) of the original SYNTAXES trial data (first row), and of the SYNTAXES trial data with additional random censoring (second row). Black dots represent Kaplan–Meier estimates in 10 equally sized groups with 0.95 confidence intervals. The shaded areas are 0.95 confidence bands of the LOESScd estimates.

### 3.2 Simulation Study

In simulated data with random censoring, the LOESScd‐based calibration curves were on average close to the true calibration curves of 10‐year mortality predictions according to the SYNTAX SCORE 2020 (Figure 4), especially in simulation scenarios of perfect calibration (Figure 4A), overestimation (Figure 4B), and overfitting (Figure 4C). In the simulation scenario of non‐linear miscalibration (Figure 4D), the LOESScd‐based calibration curve did not fully capture the true calibration curve for patients at the higher end of the predicted mortality distribution, but the bias was still limited to a mean absolute bias of 0.004 and a 0.95 quantile of the absolute bias of 0.022 (Table 1A). The bias was very similar when a spline function was used—unnecessarily—to model the relationship between predicted mortality and the censoring probability (Table 1B). Furthermore, when the data were generated with censoring increasing with risk, results were again very similar, provided that a spline function was used to model the relationship between predicted mortality and the censoring probability (Figure 5; Table 1C). The coverage of LOESScd assuming random censoring was close to nominal (Table 1A) and somewhat conservative when a relationship between predicted mortality and the censoring probability was estimated (Table 1B,C).

**FIGURE 4:**
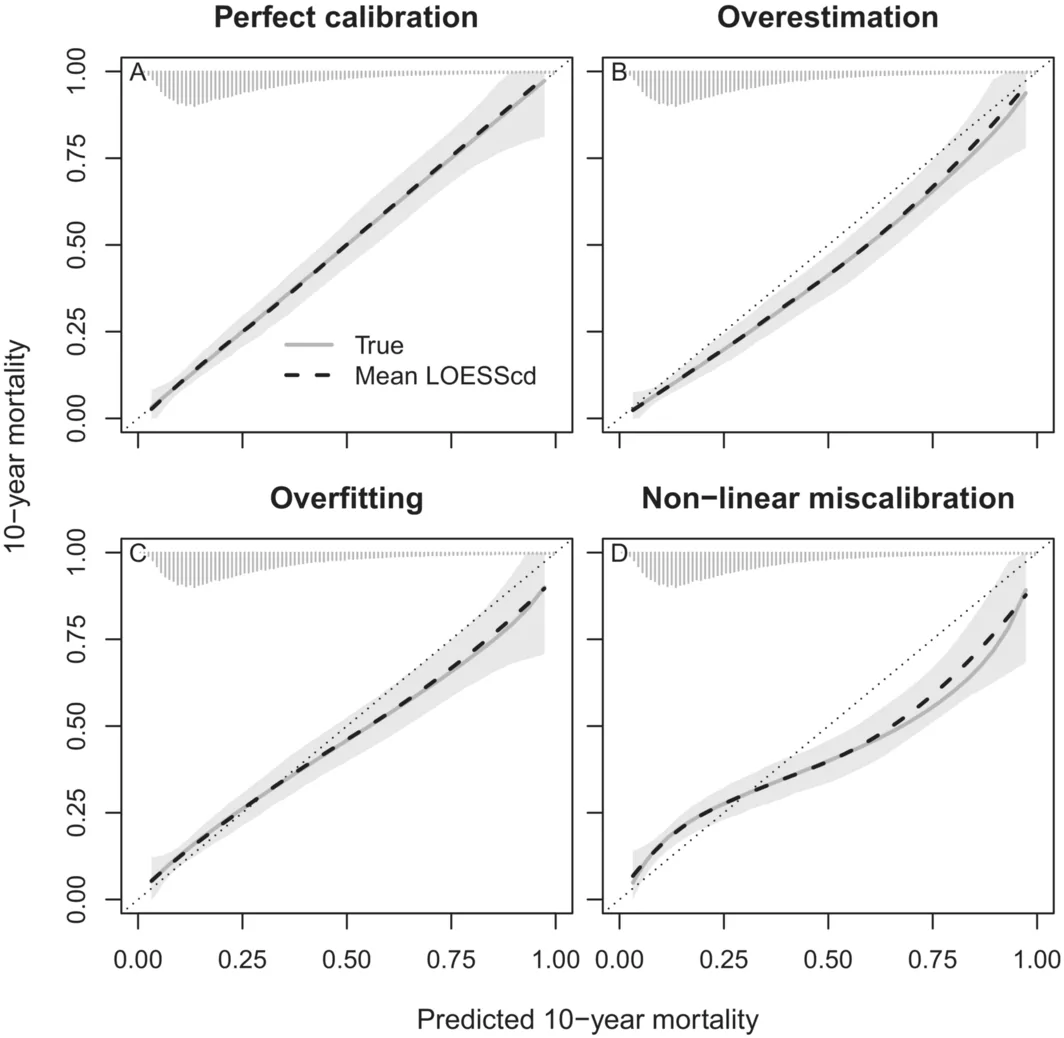
Calibration curves of 10‐year mortality predictions according to the SYNTAX SCORE 2020 in simulated data with random censoring. Simulation scenarios represent: perfect calibration (Panel A); overestimation (Panel B); overfitting (Panel C); and non‐linear miscalibration (Panel D). In all four simulation scenarios the mean LOESScd is close to the relationship between predicted and true 10‐year mortality. On top of each plot is a histogram of the distribution of predicted 10‐year mortality.

**TABLE 1:** Finite‐sample behavior of the LOESScd estimator.

	Absolute bias across 200 quantiles				
	Mean	0.75 quantile	0.95 quantile	Maximum	Coverage
A. Data with random censoring; LOESScd assumes random censoring					
Perfect calibration	0.003	0.003	0.005	0.009	0.951
Overestimation	0.002	0.002	0.008	0.027	0.950
Overfitting	0.003	0.003	0.006	0.018	0.949
Nonlinear miscalibration	0.004	0.004	0.022	0.051	0.950
B. Data with random censoring; LOESScd assumes risk‐dependent censoring					
Perfect calibration	0.002	0.003	0.004	0.005	0.957
Overestimation	0.002	0.002	0.004	0.014	0.958
Overfitting	0.002	0.003	0.004	0.013	0.955
Non‐linear miscalibration	0.004	0.003	0.021	0.047	0.956
C. Data with censoring increasing with risk; LOESScd assumes risk‐dependent censoring					
Perfect calibration	0.003	0.004	0.005	0.025	0.963
Overestimation	0.002	0.002	0.006	0.032	0.963
Overfitting	0.002	0.004	0.004	0.050	0.961
Nonlinear miscalibration	0.003	0.003	0.018	0.074	0.961

*Note:* The absolute bias at each of the 200 quantiles of the true 10‐year mortality distribution is the absolute difference between true values and the mean of the estimates across 2000 samples. Coverage is the proportion of 0.95 confidence band estimates that cover the true value across 200 quantiles and 2000 samples.

**FIGURE 5:**
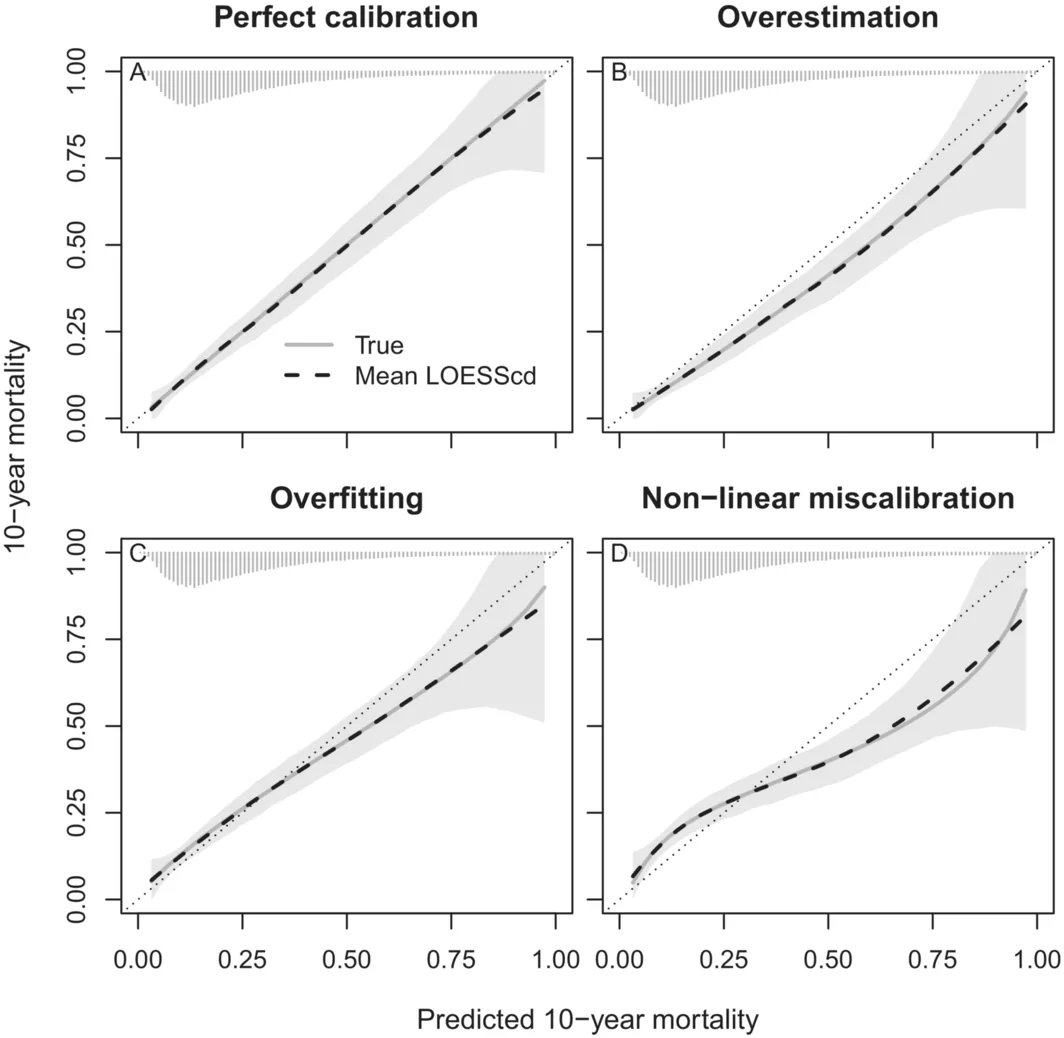
Calibration curves of 10‐year mortality predictions according to the SYNTAX SCORE 2020 in simulated data with censoring that increases with predicted mortality. Simulation scenarios represent: perfect calibration (Panel A); overestimation (Panel B); overfitting (Panel C); and non‐linear miscalibration (Panel D). In all four simulation scenarios, the mean LOESScd is close to the relationship between predicted and true 10‐year mortality. On top of each plot is a histogram of the distribution of predicted 10‐year mortality.

## 4 Discussion

LOESScd is a straightforward nonparametric estimator of the smooth relationship between a continuous covariate and the probability of an event within a specified time horizon when right‐censoring is present. LOESScd requires neither parametric assumptions on this relationship nor arbitrary binning of observations. LOESScd estimates closely follow grouped Kaplan–Meier estimates in several case study examples, with a user‐defined smoothing span to balance the ability to estimate local trends against the risk of overfitting to the data sample. LOESScd behaved well in simulations of typical prognostic model validation scenarios, showing limited bias and close‐to‐nominal coverage.

LOESScd is a valuable method for use in applied studies focused on the relationship between a continuous covariate and the probability of an event in the presence of right‐censoring. Right‐censoring of data is often encountered in the medical field, but analyses of times to a specific event are also found in other fields, for example in reliability studies where not all parts fail within the time horizon of the study [24]. In the medical field, LOESScd is particularly useful for estimation of smooth calibration curves when validating prognostic models—either traditional regression or modern AI models—in censored data [2, 3, 14]. Furthermore, LOESScd can play a role in predictive personalized medicine for nonparametric estimation of the relationship of covariates with the difference in the probability of an event across interventions [25, 26, 27]. Future research should extend LOESScd to settings with competing events [28].

LOESScd is in essence based on a censoring unbiased transformation of the outcome indicator at a specific timepoint, that is, the IPC multiplied by the outcome indicator [9, 10, 11, 12, 13]. The use of censoring unbiased transformations and/or IPC weights to estimate smooth associations between covariates and the probability of an event in the presence of right‐censoring may not be entirely novel but is almost exclusively present in the methodological literature and relatively unknown in the applied (medical) literature. Thus, part of our work serves a translational goal—to promote the use of this method in applied clinical studies, and especially for estimation of smooth calibration curves when validating prognostic models.

The advantage of LOESScd over (semi‐)parametric alternatives is that it does not require assumptions on the relationship between the continuous covariate and the probability of an event or on other model assumptions, for example, proportional hazards when using Cox regression [14]. Semi‐parametric models or parametric models may be statistically more efficient with smaller datasets if those assumptions are correct, but the performance of (semi)parametric models may degrade if the underlying assumptions are wrong. Our simulations showed good behavior of LOESScd in realistic scenarios but were not designed to examine its statistical properties under all possible circumstances. Future research should further evaluate its statistical properties in comparison with parametric alternatives, in scenarios where parametric model assumptions hold and in scenarios where model assumptions are violated.

LOESScd is especially straightforward when the probability of censoring does not depend on the continuous covariate for which we estimate the relationship with the probability of an event. In that case we use non‐parametric reverse Kaplan–Meier estimates to weigh the events. The previously proposed smoothing of pseudo‐observations would also be applicable under these circumstances [3, 15]. However, when the probability of censoring depends on the continuous covariate of interest, we need to estimate their relationship. We used a flexible Cox regression with penalized splines to estimate the probability of censoring dependent on the continuous covariate [20]. A likelihood ratio test of this Cox model against a null model can inform us if it is necessary to model the censoring pattern. Compared to estimates based on the reverse Kaplan–Meier, this method led to improved estimates in the presence of censoring that increased with the continuous covariate, and to similar estimates when censoring was random. Although we made parametric assumptions to model the censoring pattern, it would be attractive to also estimate the censoring pattern non‐parametrically, for example, with a smoothed moving average of overlapping‐window Kaplan–Meier estimates using the movStats function in Hmisc R‐package [29]. The different options for defining the censoring probability model could be part of future comparisons of LOESScd with (semi‐)parametric alternatives.

We used two smoothing spans in this paper, that is, the default smoothing span of the LOESS R‐function (0.75) and a much weaker smoothing span (0.25) to illustrate that the LOESScd is a good local approximation of Kaplan–Meier estimates in small groups of observations. Other work has already been dedicated to optimization of the smoothing span [30, 31]. These optimization procedures were out of the scope of this paper and could be a topic of further research, especially when comparing LOESScd with (semi‐)parametric alternatives.

## 5 Conclusion

In conclusion, LOESScd is a promising nonparametric estimator of the relationship of a continuous covariate with the probability of an event in the presence of right‐censoring. The method is especially useful for estimating calibration curves when validating prognostic models and avoids arbitrary binning of predicted values.

## Funding

The authors have nothing to report.

## Conflicts of Interest

The authors declare no conflicts of interest.

## Data Availability

The code and simulation data used in this project are available in the public GitLab repository https://gitlab.com/davidvanklaveren/paper_loess_cd. The original SYNTAXES data are available from the corresponding author upon reasonable request.

## References

[1] W. S. Cleveland , “Robust Locally Weighted Regression and Smoothing Scatterplots,” Journal of the American Statistical Association 74, no. 368 (1979): 829–836, 10.2307/2286407.

[2] P. C. Austin and E. W. Steyerberg , “Graphical Assessment of Internal and External Calibration of Logistic Regression Models by Using Loess Smoothers,” Statistics in Medicine 33, no. 3 (2014): 517–535, 10.1002/sim.5941. PMC4793659 24002997

[3] R. D. Riley , L. Archer , K. I. E. Snell , et al., “Evaluation of Clinical Prediction Models (Part 2): How to Undertake an External Validation Study,” BMJ 384 (2024): e074820, 10.1136/bmj-2023-074820. PMC10788734 38224968

[4] National Institute of Standards and Technology , “e‐Handbook of Statistical Methods—Chapter 4.1.4.4,” (2025), https://www.itl.nist.gov/div898/handbook/pmd/section1/pmd144.html.

[5] T. G. Clark , M. J. Bradburn , S. B. Love , and D. G. Altman , “Survival Analysis Part I: Basic Concepts and First Analyses,” British Journal of Cancer 89, no. 2 (2003): 232–238, 10.1038/sj.bjc.6601118. PMC2394262 12865907

[6] E. L. Kaplan and P. Meier , “Nonparametric Estimation From Incomplete Observations,” Journal of the American Statistical Association 53, no. 282 (1958): 457–481, 10.2307/2281868.

[7] D. R. Cox , “Regression Models and Life‐Tables,” Journal of the Royal Statistical Society: Series B: Methodological 34, no. 2 (1972): 187–202, 10.1111/j.2517-6161.1972.tb00899.x.

[8] C. Kooperberg , C. J. Stone , and Y. K. Truong , “Hazard Regression,” Journal of the American Statistical Association 90, no. 429 (1995): 78–94, 10.2307/2291132.

[9] H. Koul , V. Susarla , and J. Van Ryzin , “Regression Analysis With Randomly Right‐Censored Data,” Annals of Statistics 9, no. 6 (1981): 1276–1288, 10.1214/aos/1176345644.

[10] J. Fan and I. Gijbels , “Censored Regression: Local Linear Approximations and Their Applications,” Journal of the American Statistical Association 89, no. 426 (1994): 560–570, 10.1080/01621459.1994.10476781.

[11] D. Rubin and M. J. van der Laan , “A Doubly Robust Censoring Unbiased Transformation,” International Journal of Biostatistics 3, no. 1 (2007): 4, 10.2202/1557-4679.1052. 22550646

[12] J. A. Steingrimsson , L. Diao , A. M. Molinaro , and R. L. Strawderman , “Doubly Robust Survival Trees,” Statistics in Medicine 35, no. 20 (2016): 3595–3612, 10.1002/sim.6949. PMC7286558 27037609

[13] D. M. Vock , J. Wolfson , S. Bandyopadhyay , et al., “Adapting Machine Learning Techniques to Censored Time‐To‐Event Health Record Data: A General‐Purpose Approach Using Inverse Probability of Censoring Weighting,” Journal of Biomedical Informatics 61 (2016): 119–131, 10.1016/j.jbi.2016.03.009. PMC4893987 26992568

[14] P. C. Austin , F. E. Harrell, Jr. , and D. van Klaveren , “Graphical Calibration Curves and the Integrated Calibration Index (ICI) for Survival Models,” Statistics in Medicine 39, no. 21 (2020): 2714–2742, 10.1002/sim.8570. PMC7497089 32548928

[15] P. Royston , “Tools for Checking Calibration of a Cox Model in External Validation: Approach Based on Individual Event Probabilities,” Stata Journal 14, no. 4 (2014): 738–755, 10.1177/1536867X1401400403.

[16] G. A. Satten and S. Datta , “The Kaplan–Meier Estimator as an Inverse‐Probability‐Of‐Censoring Weighted Average,” American Statistician 55, no. 3 (2001): 207–210, 10.1198/000313001317098185. PMC5568678 28845048

[17] F. W. Mohr , M. C. Morice , A. P. Kappetein , et al., “Coronary Artery Bypass Graft Surgery Versus Percutaneous Coronary Intervention in Patients With Three‐Vessel Disease and Left Main Coronary Disease: 5‐Year Follow‐Up of the Randomised, Clinical SYNTAX Trial,” Lancet 381, no. 9867 (2013): 629–638, 10.1016/s0140-6736(13)60141-5. 23439102

[18] D. Thuijs , A. P. Kappetein , P. W. Serruys , et al., “Percutaneous Coronary Intervention Versus Coronary Artery Bypass Grafting in Patients With Three‐Vessel or Left Main Coronary Artery Disease: 10‐Year Follow‐Up of the Multicentre Randomised Controlled SYNTAX Trial,” Lancet 394, no. 10206 (2019): 1325–1334, 10.1016/s0140-6736(19)31997-x. 31488373

[19] K. Takahashi , P. W. Serruys , V. Fuster , et al., “Redevelopment and Validation of the SYNTAX Score II to Individualise Decision Making Between Percutaneous and Surgical Revascularisation in Patients With Complex Coronary Artery Disease: Secondary Analysis of the Multicentre Randomised Controlled SYNTAXES Trial With External Cohort Validation,” Lancet 396, no. 10260 (2020): 1399–1412, 10.1016/s0140-6736(20)32114-0. 33038944

[20] P. H. C. Eilers and B. D. Marx , “Flexible Smoothing With B‐Splines and Penalties,” Statistical Science 11, no. 2 (1996): 89–121, 10.1214/ss/1038425655.

[21] H. Demirtas and B. Doganay , “Simultaneous Generation of Binary and Normal Data With Specified Marginal and Association Structures,” Journal of Biopharmaceutical Statistics 22, no. 2 (2012): 223–236, 10.1080/10543406.2010.521874. 22251171

[22] “BinNor: Simultaneous Generation of Multivariate Binary and Normal,” (2025), https://CRAN.R‐project.org/package=BinNor. Version R package version 2.3.4.

[23] T. P. Morris , I. R. White , and M. J. Crowther , “Using Simulation Studies to Evaluate Statistical Methods,” Statistics in Medicine 38, no. 11 (2019): 2074–2102, 10.1002/sim.8086. PMC6492164 30652356

[24] W. J. C. Verhagen and L. W. M. De Boer , “Predictive Maintenance for Aircraft Components Using Proportional Hazard Models,” Journal of Industrial Information Integration 12 (2018): 23–30, 10.1016/j.jii.2018.04.004.

[25] M. Bonetti and R. D. Gelber , “A Graphical Method to Assess Treatment‐Covariate Interactions Using the Cox Model on Subsets of the Data,” Statistics in Medicine 19, no. 19 (2000): 2595–2609, 10.1002/1097-0258(20001015)19:19<2595::aid-sim562>3.0.co;2-m. 10986536

[26] D. M. Kent , E. Steyerberg , and D. van Klaveren , “Personalized Evidence Based Medicine: Predictive Approaches to Heterogeneous Treatment Effects,” BMJ 363 (2018): k4245, 10.1136/bmj.k4245. PMC6889830 30530757

[27] E. M. Trinks‐Roerdink , G. J. Geersing , C. J. van den Dries , et al., “Integrated Care in Patients With Atrial Fibrillation–A Predictive Heterogeneous Treatment Effect Analysis of the ALL‐IN Trial,” PLoS One 18, no. 10 (2023): e0292586, 10.1371/journal.pone.0292586. PMC10586661 37856486

[28] P. C. Austin , H. Putter , D. Giardiello , and D. van Klaveren , “Graphical Calibration Curves and the Integrated Calibration Index (ICI) for Competing Risk Models,” Diagnostic and Prognostic Research 6, no. 1 (2022): 2, 10.1186/s41512-021-00114-. PMC8762819 35039069

[29] “Hmisc: Harrell Miscellaneous. R Package Version 5.2–5,” (2026), http://CRAN.R‐project.org/package=Hmisc.

[30] D. Ruppert , S. J. Sheather , and M. P. Wand , “An Effective Bandwidth Selector for Local Least Squares Regression,” Journal of the American Statistical Association 90, no. 432 (1995): 1257–1270, 10.2307/2291516.

[31] C. M. Hurvich , J. S. Simonoff , and C.‐L. Tsai , “Smoothing Parameter Selection in Nonparametric Regression Using an Improved Akaike Information Criterion,” Journal of the Royal Statistical Society. Series B, Statistical Methodology 60, no. 2 (2002): 271–293, 10.1111/1467-9868.00125.

[32] “A Package for Survival Analysis in R. R Package Version 3.8‐3,” (2024), https://CRAN.R‐project.org/package=survival.

